# Laparoscopic repair for interparietal hernia after enhanced-view totally extraperitoneal hernia repair: A case report

**DOI:** 10.1016/j.ijscr.2023.108552

**Published:** 2023-07-21

**Authors:** Shusaku Honma, Takashi Takashima, Tatsuhi Ushikubo, Kana Ishikawa, Takahisa Suzuki, Sanae Nakajima

**Affiliations:** Department of Surgery, Kobe City Medical Center West Hospital, 2-4, Ichibancho, Nagataku, Kobe, Hyogo 653-0013, Japan

**Keywords:** Interparietal hernia, eTEP, Complication, Ventral hernia

## Abstract

**Introduction:**

The enhanced-view totally extraperitoneal (eTEP) technique, an endoscopically performed Rives-Stoppa method, has been used extensively for ventral hernia repairs. However, in this technique, the necessity of posterior rectus sheath re-approximation and mesh fixation remains unclear. There are a few reports of post-eTEP interparietal hernias (IHs) occurring because of dehiscence of the re-approximated posterior rectus sheath; however, IH secondary to mesh migration is rare. Herein, we report a rare case of IH due to mesh migration after eTEP repair for an incisional hernia.

**Presentation of case:**

A 70-year-old man underwent eTEP repair for an incisional hernia using a self-gripping mesh without mesh fixation and posterior rectus sheath re-approximation one year previously, developed an IH. An elective laparoscopic surgery revealed an orifice to the retrorectus space as though the IH sac between the retrorectus muscle and the posterior layer including the bilateral posterior rectus sheaths, peritoneum, and mesh. We placed eight transmural sutures with 0 nylon thread and closed the orifice. The patient was then discharged on postoperative day two and was asymptomatic at 24 months without evidence of ventral hernia recurrence.

**Discussion:**

We consider that strenuous activity and deep bending may cause mesh migration or dislocation. If that occurs in the early post-eTEP period without posterior rectus sheaths closure, the vulnerable peritoneal area will be exposed, which consider to be an IH orifice.

**Conclusions:**

Even after using the self-gripping mesh in eTEP repair, mesh fixation remains the best option to prevent postoperative complications, including IH.

## Highlights

• The eTEP technique is used extensively for ventral hernia repairs.
• However, the necessity of posterior rectus sheath re-approximation remains unclear.
• We report a rare case of IH due to mesh migration after a previous eTEP repair.
• The patient was asymptomatic at 24 months without ventral hernia recurrence after IH repair.
• Mesh fixation remains optimal for preventing postoperative complications.

## 1 Introduction

Since Miserez first described the enhanced-view totally extraperitoneal (eTEP) technique in 2002, this endoscopically performed Rives-Stoppa technique has been widely used for ventral hernia repair [1,2]. This technique avoids mesh-and-tacker-related complications such as mesh erosion, adhesive bowel obstruction, and chronic pain, which are common characteristics of the laparoscopic intraperitoneal onlay mesh technique, by placing the mesh in the retrorectus space [3]. Retromuscular ventral hernia repair, including that performed with the eTEP technique, has a unique complication: an interparietal hernia (IH) resulting from dehiscence of the posterior rectus sheath [4,5]. Most patients with this condition present with bowel obstruction and require emergency surgical treatment in the early postoperative period. However, in the eTEP technique, the necessity of posterior rectus sheath re-approximation and mesh fixation remains unclear [2,[6], [7], [8]]. At our hospital, neither posterior rectus sheath re-approximation nor mesh fixation has been performed for eTEP repair, but we encountered an IH case one year after surgery. Here, we report a rare case of IH that was considered due to mesh migration after eTEP repair for an incisional hernia instead of dehiscence of the posterior layer. This case has been reported in line with the SCARE criteria [9].

## 2 Case presentation

**Fig. 1:**
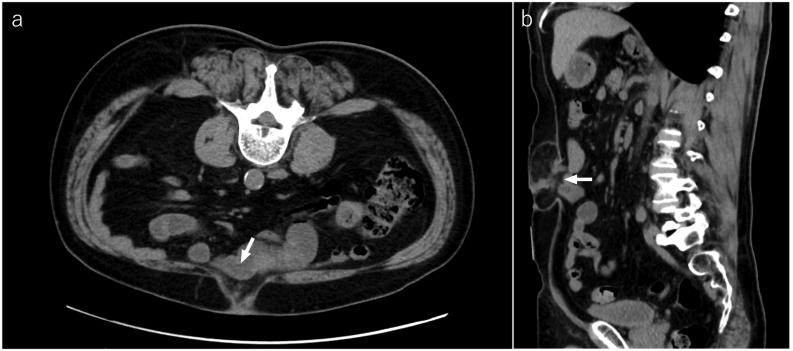
Abdominal computed tomography in the prone position before enhanced-view totally extraperitoneal repair demonstrates an incisional hernial orifice in the umbilicus. a) Axial image showing a hernia defect measuring 5 cm in width (arrow). b) Sagittal image showing a hernia defect measuring 4 cm in length (arrow).

**Fig. 2:**
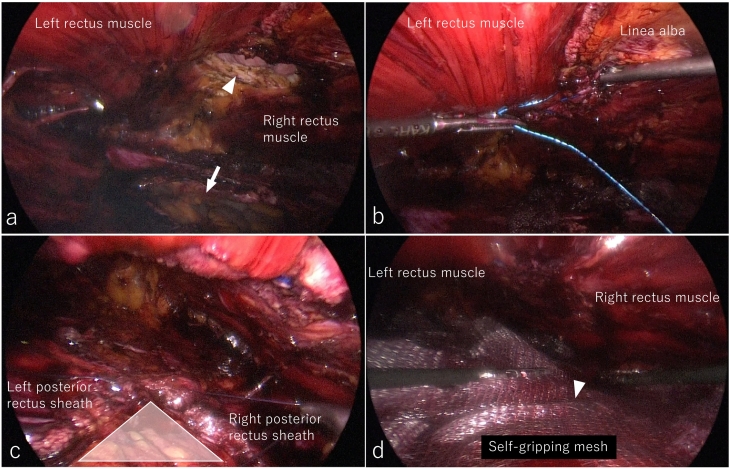
Intraoperative endoscopic views in enhanced-view totally extraperitoneal repair. a) The retrorectus and preperitoneal spaces were dissected from cranial to caudal direction, incising bilateral posterior rectus sheaths. The arrowhead and arrow show the abdominal and the peritoneum defects, respectively. b) The abdominal defect was closed with 0 non-absorbable barbed sutures. c) The peritoneum defect was closed with 3-0 absorbable sutures. The triangle zone indicates the vulnerable peritoneal area between the left and right posterior rectus sheaths. d) A 20-cm-long and 15-cm-wide self-gripping mesh was placed in the retrorectus space with no fixation. Arrowhead indicates the center of the mesh.

A 70-year-old man underwent laparoscopic ventral hernia repair with the eTEP technique for an incisional hernia after laparoscopic cholecystectomy (Fig. 1). During the procedure, a 4 cm long and 5 cm wide hernia defect was found at the umbilicus. Bilateral retrorectus and preperitoneal dissection were then performed in the caudal direction, incising the bilateral posterior rectus sheaths to the hernia defect. The hernial sac was then dissected, and bilateral retrorectus dissection was continued to the pubis level. After the development of sufficient preperitoneal and bilateral retrorectus spaces, the abdominal and peritoneal defects were closed with 0 non-absorbable barbed and 3–0 absorbable sutures, respectively. However, the posterior rectus sheath could not be re-approximated. Finally, a 20-cm-long and 15-cm-wide self-gripping mesh was placed in the retrorectus space in which grips were on the peritoneum side with no fixation (Fig. 2). We confirmed that the upper edge of the mesh reached the cranial margin of the dissection space and the center of the mesh was approximately at the umbilicus. The patient was then discharged on postoperative day three and remained symptom-free.

**Fig. 3:**
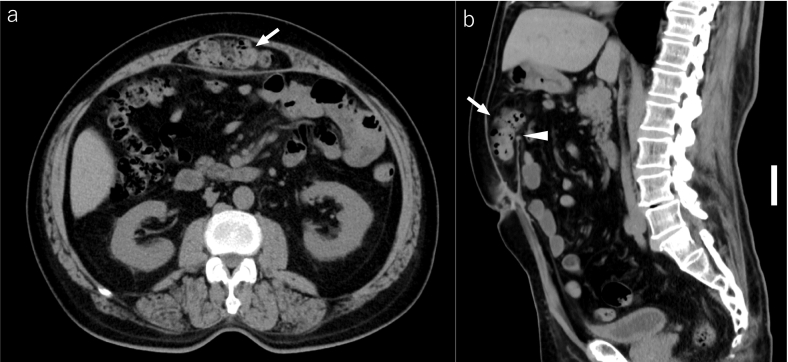
Abdominal computed tomography one year after enhanced-view totally extraperitoneal repair. a) Axial image shows the large bowel (arrow) in the retrorectus space. b) Sagittal image shows the large bowel (arrow) entering the defect of the posterior rectus sheath. An arrowhead indicates the upper edge of the mesh. There is no evidence of bowel obstruction and dehiscence of the anterior rectus sheaths. Scale bar, 5 cm.

**Fig. 4:**
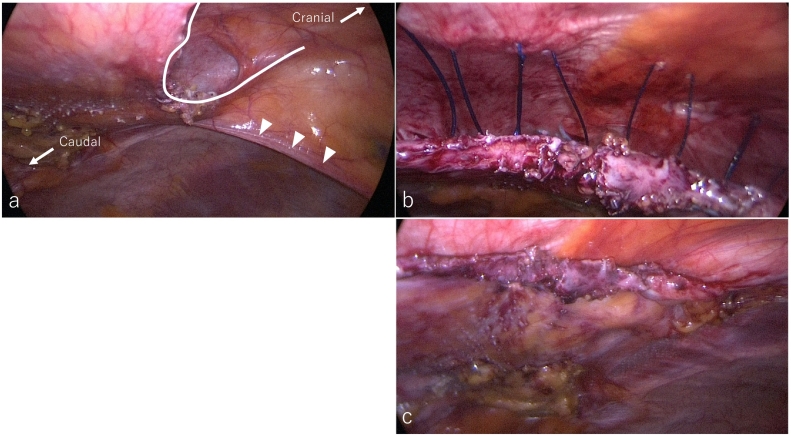
Intraoperative endoscopic views in interparietal hernia repair. a) After removing the adhesion between the abdominal wall and the omentum. Dots line and arrowheads indicate interparietal hernia defect and round ligament of the liver, respectively. b) The interparietal hernia defect was closed with 0 nylon thread transmural stitches. c) After closing the orifice into the retrorectus space. b) and c) images were both cranial to caudal view.

Abdominal computed tomography (CT) was taken during the follow-up visit one year after the surgery. It demonstrated herniation of the large bowel into the space between the rectus muscle and the posterior layer (Fig. 3). We suspected that the mesh had migrated because it was only 6 cm from the umbilicus to the upper edge of the mesh. Although there was no evidence of bowel obstruction, we also suspected that the peritoneal breakdown occurred, and the large bowel was in contact with the mesh, which could lead its erosion into the large bowel. Because the patient was asymptomatic, we performed elective laparoscopic surgery, which revealed a cavity between the rectus muscle and the posterior layer including the bilateral posterior rectus sheaths, peritoneum, and mesh in the epigastric abdominal wall, confirming the diagnosis of an IH. No adhesion was present between the internal organ and the IH sac, despite slight omental adhesions around the IH defect. We performed eight transmural stitches with 0 nylon thread and closed the orifice to the retrorectus space (Fig. 4). The operating time was 116 min, and the amount of bleeding was minimal and hence could not be measured.

**Fig. 5:**
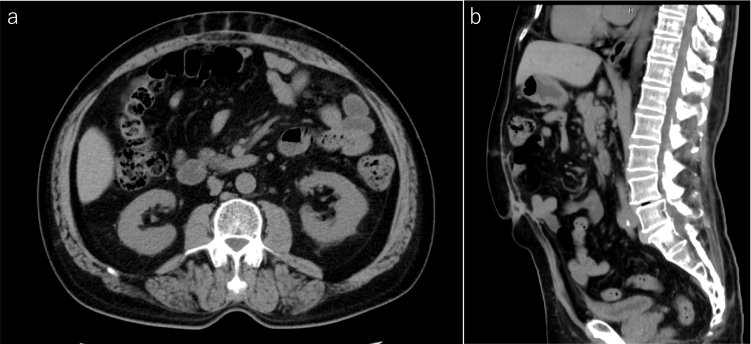
Abdominal computed tomography 6 months after interparietal hernia repair. There was no evidence of hernia recurrence, including interparietal hernias. a) and b) show the axial and sagittal images, respectively.

There were no postoperative complications, and the patient was discharged on postoperative day two. No hernia recurrence, including IH, occurred after 24 months of follow-up (Fig. 5).

## 3 Discussion

IHs are defined as protrusions of intra-abdominal contents within the layers of the abdominal wall, which rarely occur as postoperative complications [10,11]. Only a few reports have described postoperative IHs occurring as a result of dehiscence of the posterior rectus sheath closure after retromuscular ventral hernia repair, including the eTEP technique [4,5,12]. In the eTEP technique, we incise the medial portion of the bilateral posterior rectus sheaths, and then the bilateral retrorectus and preperitoneal spaces are connected. After the creation of the retromuscular space, we can place the mesh in this space; however, the necessity of posterior rectus sheath re-approximation and mesh fixation remains controversial, as opposed to the fact that the hernia defect should be closed [2,[6], [7], [8]]. Li et al. reported that the posterior rectus sheaths should be closed to reinforce the abdominal wall [8]. However, when closing the posterior rectus sheath, excessive tension on the posterior layer may be a problem, which could lead to a breakdown in the posterior layer in the early postoperative period [4,5]. In contrast, Schwarz et al. suggested that closure of the posterior rectus sheath is not necessary for the success of the eTEP technique. They proposed that restoration of the linea alba by suturing the anterior sheaths of the rectus muscles, which improves the isokinetic and isometric functions of the abdominal wall and eventually the quality of life, was the goal of abdominal wall reconstruction [2,6]. They also reported that closure of the peritoneal defect is only to maintain a barrier between the mesh and the intra-abdominal contents and not to be a resistance layer [2,6]. Therefore, if the mesh migration or dislocation occurs in the early post-eTEP period without posterior rectus sheaths closure, the vulnerable peritoneal area between the left and right posterior rectus sheaths will be exposed, which consider to be an IH orifice.

Radu et al. reported that mesh fixation was unnecessary because the retromuscular space was restricted, resulting in a low possibility of mesh migration [2]. Furthermore, fixation was considered irrelevant when using a self-gripping mesh. A self-gripping mesh that does not require any fixation has been used in inguinal and ventral hernia repair, in which mesh migration is not a concern, to avoid acute and chronic pain caused by tacks or transfascial sutures [[13], [14], [15]]. In our case, from intraoperative findings at the second operation, mesh migration was considered to be the reason for IH despite using the self-gripping mesh. Although the mechanisms and factors of mesh migration or dislocation remain unknown, strenuous activity and deep bending may cause mesh migration or dislocation, especially soon after surgery, a period during which the mesh has the greatest possibility of movement [16]. Our patient returned to work, which was deemed to be a strenuous activity a week after surgery, which seems to lead to mesh migration or dislocation. We consider that covering the entire dissected area by mesh in addition to minimum necessary mesh fixation by some method is important to avoid mesh migration or dislocation in the early post-eTEP period when posterior rectus sheath re-approximation is not performed.

## 4 Conclusion

Although the necessity of mesh fixation in eTEP repair remains controversial, we consider that mesh fixation, even after using the self-gripping mesh, in a few points along the abdominal wall is one option to prevent the incidence of IH in the early post-eTEP period.

## Consent

Written informed consent was obtained from the patient for publication of this case report and accompanying images. A copy of the written consent is available for review by the Editor-in-Chief of this journal on request.

## Ethical approval

No ethical approval was necessary because case reports are exempted from ethical approval in our institution, Kobe City Medical Center West Hosipital, Hyogo, Japan.

## Funding

This study did not receive any funding.

## Author contribution

Shusaku Honma contributed to the study concept, data collection, writing the paper. Shusaku Honma and Kana Ishikawa performed the surgery. Takashi Takashima and Tatsuhi Ushikubo followed up the patient. Takahisa Suzuki and Sanae Nakajima contributed to writing the paper.

## Guarantor

Shusaku Honma is the guarantor of this work.

## Registration of research studies

Not required for this report.

## Declaration of competing interest

All authors declare that they have no competing interests.

## Data Availability

The datasets supporting the conclusions of this article are included in this paper.
